# Quantitative analysis and evaluation of research on the application of computer vision in sports since the 21st century

**DOI:** 10.3389/fspor.2025.1604232

**Published:** 2025-07-15

**Authors:** Cheng Chen, Jiaxin Xue, Wenling Gou, Mengning Xie, Xiaolin Yao

**Affiliations:** ^1^ Graduate School, Harbin Sport University, Harbin, China; ^2^ School of Physical Education, East China University of Technology, NanChang, China; ^3^ Faculty of Physical and Sports Sciences and Techniques, University of Montpellier, Montpellier, France; ^4^ Institute of Sports Humanities and Society, Harbin Sport University, Harbin, China

**Keywords:** sports, computer vision, artificial intelligence, health monitoring, injury prevention, referee assistance

## Abstract

**Introduction:**

Integrating computer vision with sports has significantly transformed competitive, educational, and recreational sports practices. A review of the literature in this field is imperative. The purpose of this paper is to reveal the field's temporal, disciplinary, geographic, journal, and collaborative characteristics and summarize research themes and future trends to promote a systematic understanding of the field within the academic community.

**Methods:**

To identify research trends, a bibliometric analysis of 1,209 publications retrieved from the Science Citation Index Expanded in the Web of Science core database was conducted.

**Results:**

In terms of time series, publications in the field grew slowly until 2014, and publications in the field increased significantly after 2015, with polynomial models predicting 206, 233, and 263 annual publications over the next three years. In terms of disciplinary structure, three frontier disciplines utilized interdisciplinary knowledge provided by four basic disciplines to make cutting-edge breakthroughs. Geospatially, there is a three-way split between China, the United States, and the United Kingdom, while most African countries are not involved in the research. Regarding journal distribution, research in this field was published in five Q2 and four Q1 journals, mainly in computing, with fewer contributions from sports journals. The study identifies five principal research themes: skill optimization, health monitoring and injury prevention, physical performance assessment, tactical analysis and referee assistance, and immersive event experiences. Furthermore, it highlights existing research limitations and outlines directions for future exploration.

**Conclusions:**

Computer vision research in sports has shown high explosive growth in recent years. The field is interdisciplinary but lacks collaboration among interdisciplinary research teams. The quality of the journals published is high, but the main focus is on computer-based journals. The theme of research in this field is centered on the fundamental characteristic of serving human beings.

## 1 Introduction

In the context of the technological advances of the 21st century, artificial intelligence (AI) has emerged as the core driving force behind profound changes in various fields of society (1). Computer vision (CV), an important branch of AI, has been widely used in many industries due to its excellent image processing and analysis capabilities (2). This technology has demonstrated transformative impacts in critical fields including autonomous driving, medical imaging, and industrial inspection (3–5).

The field of sports has similarly experienced a transformative shift. Sports computer vision (SCV) has revolutionized competitive, educational, and recreational sports, propelling them into an intelligent era (6, 7). Academic research on SCV has generated substantial knowledge production. In individual sports, studies have focused on diving (8), tennis (9), badminton (10), and table tennis (11), while in team sports, research has explored basketball (12), football (13), cricket (14), volleyball (15), hockey (16), and handball (17). The field is characterized by a dual emphasis on algorithm innovation and application innovation within sports scenarios (18–20). Meanwhile, visual algorithm models are being increasingly extended to various sports-related domains, such as sports manufacturing (21), sports journalism (22), sports economics (23), and esports (24).

However, following explosive growth, SCV application research has become increasingly fragmented, creating challenges in developing a comprehensive panoramic view. This fragmentation hinders researchers' ability to form a systematic understanding of the field's knowledge network and its overall dynamics. Conducting a literature review is essential for gaining an in-depth understanding of the research area, and several scholars have conducted reviews on SCV. Thomas G. et al. (25) focused on SCV applications in commercial sports. Colyer et al. (26) examined the evolution of SCV technology in sports. Naik et al. (27) analyzed it from the perspective of sports video analysis. Kristina et al. (28) provided an overview of SCV in human action recognition, while Debnath et al. (29) evaluated it in sports rehabilitation. In summary, all of the above qualitative review studies have made substantial contributions to the academic community. However, they exhibit two primary limitations:

• Methodologically, existing studies remain predominantly one-dimensional, relying chiefly on qualitative analyses that risk introducing subjective bias into research findings (30).
• Thematically, the research landscape appears fragmented, with scholars either examining specific sports scenarios in isolation or focusing exclusively on technical dimensions, failing to provide a comprehensive, integrative perspective.

In this context, bibliometric studies offer a promising solution. By systematically mapping the knowledge structure of the field and uncovering hidden relationships and trends within the literature, bibliometric methods can effectively address the methodological limitations of existing research (31, 32). Specifically, it examines: the field's temporal trends, knowledge structure, geographical distribution, journal contributions, and research collaboration patterns. Finally, the paper discusses the main application areas, current challenges, ethical issues and potential future directions of SCV.

## 2 Materials and methods

### 2.1 Database and search strategy

This study primarily considers two key dimensions to ensure a comprehensive analysis: (1) data from the Web of Science (WOS) Core Collection were analyzed, and (2) a set of detailed selection criteria was established to identify studies specifically focused on SCV applications.

These databases were selected due to their high relevance, rigorous indexing standards, and widespread use in bibliometric research. The WOS provides comprehensive coverage of high-impact scientific literature, ensuring that the dataset used for analysis is robust, reliable, and representative of the research field (33–35). Although Scopus and SportDiscus are also commonly used databases in bibliometric research, they were not included in this study for the following reasons. First, despite Scopus's extensive journal coverage, the heterogeneity in its indexing criteria and quality thresholds introduces variability that may compromise the consistency of bibliometric metrics. This makes it difficult to ensure methodological uniformity and hampers robust comparative analysis with the more standardized dataset provided by the WOS Core Collection. Additionally, Scopus lacks support for standardized citation formats and certain advanced bibliometric tools, which may affect the accuracy of the analysis. Second, SportDiscus has the advantage of being sports-focused, but this specialization is also a limitation, as it does not fully align with the interdisciplinary nature required for this bibliometric study. Therefore, to ensure data quality, comparability, and reliability of research findings, this study exclusively selected the WOS Core Collection as the source of analysis.

Furthermore, to ensure a transparent and reproducible data collection process, the Preferred Reporting Items for Systematic Reviews and Meta-Analyses (PRISMA) guidelines were followed (36). Given the scope of this review, a broad thematic search query was employed: [TI = (“computer vision” OR “image processing” OR “image classification” OR “object detection” OR “pose estimation” OR “action recognition” OR “motion tracking” OR “image segmentation” OR “video analysis”) AND TI = (“sport*” OR “athletes” OR “exercise” OR “sports training” OR “sports performance” OR “sports biomechanics” OR “sports rehabilitation” OR “individual athlete training” OR “coaching in team sports” OR “helping coaches monitor game situations” OR “referee decision assistance” OR “post-match analysis” OR “post-game statistics and evaluation”)] OR[AB = (“computer vision” OR “image processing” OR “image classification” OR “object detection” OR “pose estimation” OR “action recognition” OR “motion tracking” OR “image segmentation” OR “video analysis”) AND AB = (“sport*” OR “athletes” OR “exercise” OR “sports training” OR “sports performance” OR “sports biomechanics” OR “sports rehabilitation” OR “individual athlete training” OR “coaching in team sports” OR “helping coaches monitor game situations” OR “referee decision assistance” OR “post-match analysis” OR “post-game statistics and evaluation”)]. This approach allowed for a systematic and replicable data extraction method. The search was conducted on January 1, 2025, covering the period from January 1, 2000, to December 31, 2024, which aligns with the historical development of SCV research. Table 1 summarizes the search strategy, search terms, Boolean operators and their combinations, as well as the rationale for the selection of search terms and logic settings.

**Table 1:** Literature search strategy.

Search Category	Keywords	Boolean Operators	Search Scope	Reason for Selection
Computer Vision Technologies	Computer vision, image processing, image classification, object detection, pose estimation, action recognition, motion tracking, image segmentation, video analysis	OR	Title/Abstract	These are core technologies in computer vision, directly related to sports analysis and training. Including these terms ensures the retrieval of literature concerning video and motion analysis.
Sports-related Terminology	Sport*, athletes, exercise, sports training, sports performance, sports biomechanics, sports rehabilitation, individual athlete training, coaching in team sports, helping coaches monitor game situations, referee decision assistance, post-match analysis, post-game statistics and evaluation	OR	Title/Abstract	These terms are closely associated with sports and physical activity, encompassing athlete training, competition analysis, and performance evaluation. Their inclusion ensures the comprehensive identification of literature on computer vision applications in sports science.
Combined Search Strategy	Computer Vision Technologies AND Sports-related Terminology	AND	Title/Abstract	The Boolean operator AND is employed to ensure the co-occurrence of computer vision technologies and sports-related terms within the title or abstract, facilitating the retrieval of literature at the intersection of both fields.

### 2.2 Data screening

**Figure 1:**
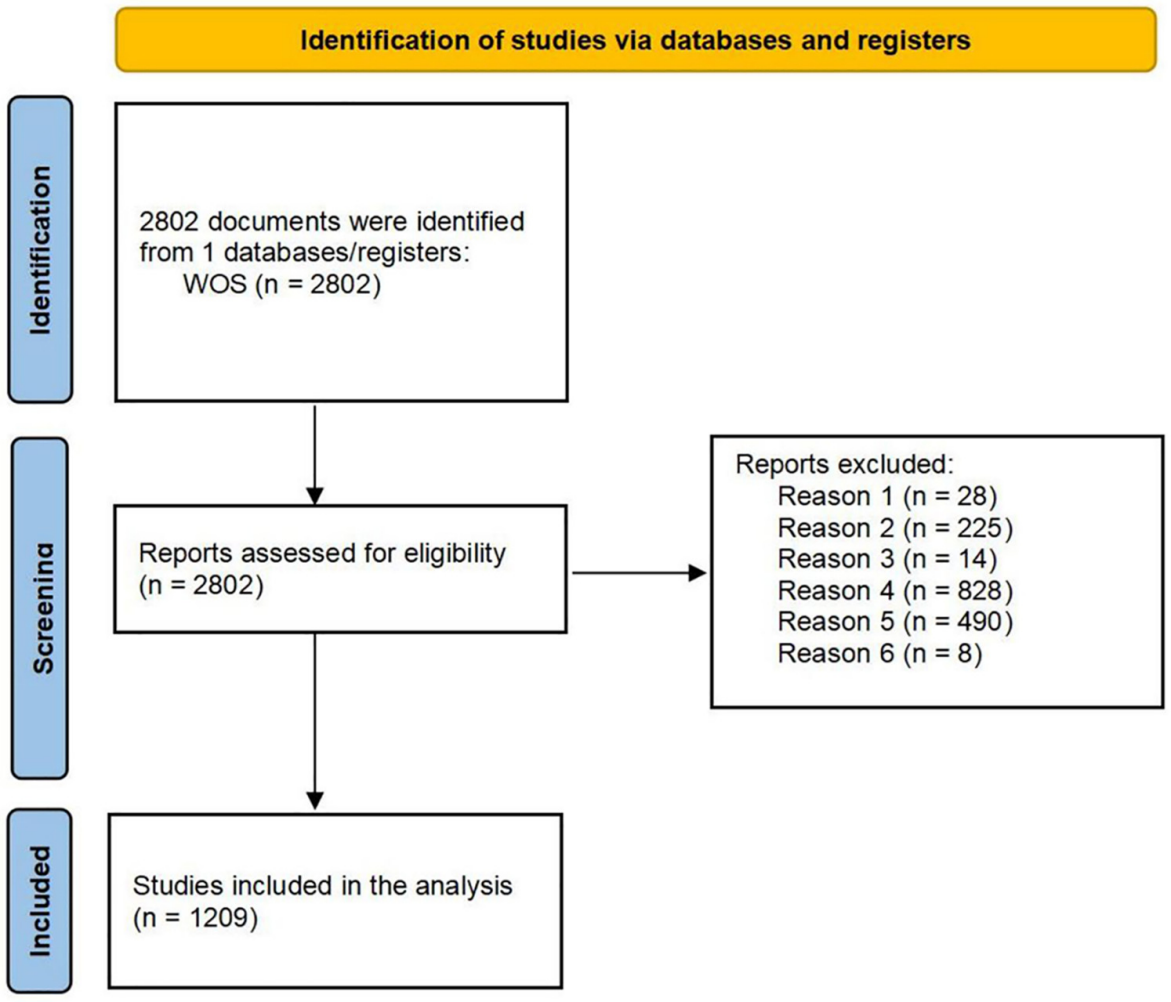
Identification of studies via PRISMA 2020 protocol.

Figure 1 shows the literature screening process. We identified 2,802 records from WOS Core Collection, followed by screening and eligibility assessment.

• Publications from the year 2025 were excluded (*n* = 28).
• Exclusion of any literature other than journals or conference papers(*n* = 225).
• Relevant studies that were not in English were excluded(*n* = 14).
• Research papers within the fields of earth sciences, agronomy, and transport that may refer to ’sport' in a different sense were excluded (*n* = 828), such as “Application of artificial neural networks for buckling prediction in functionally graded concrete sports structures and efficiency enhancement” (37).
• Export the plain-text file.
• The abstracts were read, and irrelevant literature was removed(*n* = 490). such as “Event-based High-speed Ball Detection in Sports Video” (38).
• The data was deduplicated using CiteSpace(*n* = 8).
• After the above process, 1,209 pieces of literature were included.

### 2.3 Data analysis method

To ensure the comprehensiveness of the sample and leverage the unique features of the tools, we employed a combination of Bibliometrics, SciExplorer, and CiteSpace for an in-depth bibliometric analysis of the field. Based on their respective strengths, Bibliometrix was used for time series and collaboration network analysis, SciExplorer for geographical and journal distribution analysis, and Cite Space for knowledge structure analysis and keyword clustering discussions.

## 3 Results

**Figure 2:**
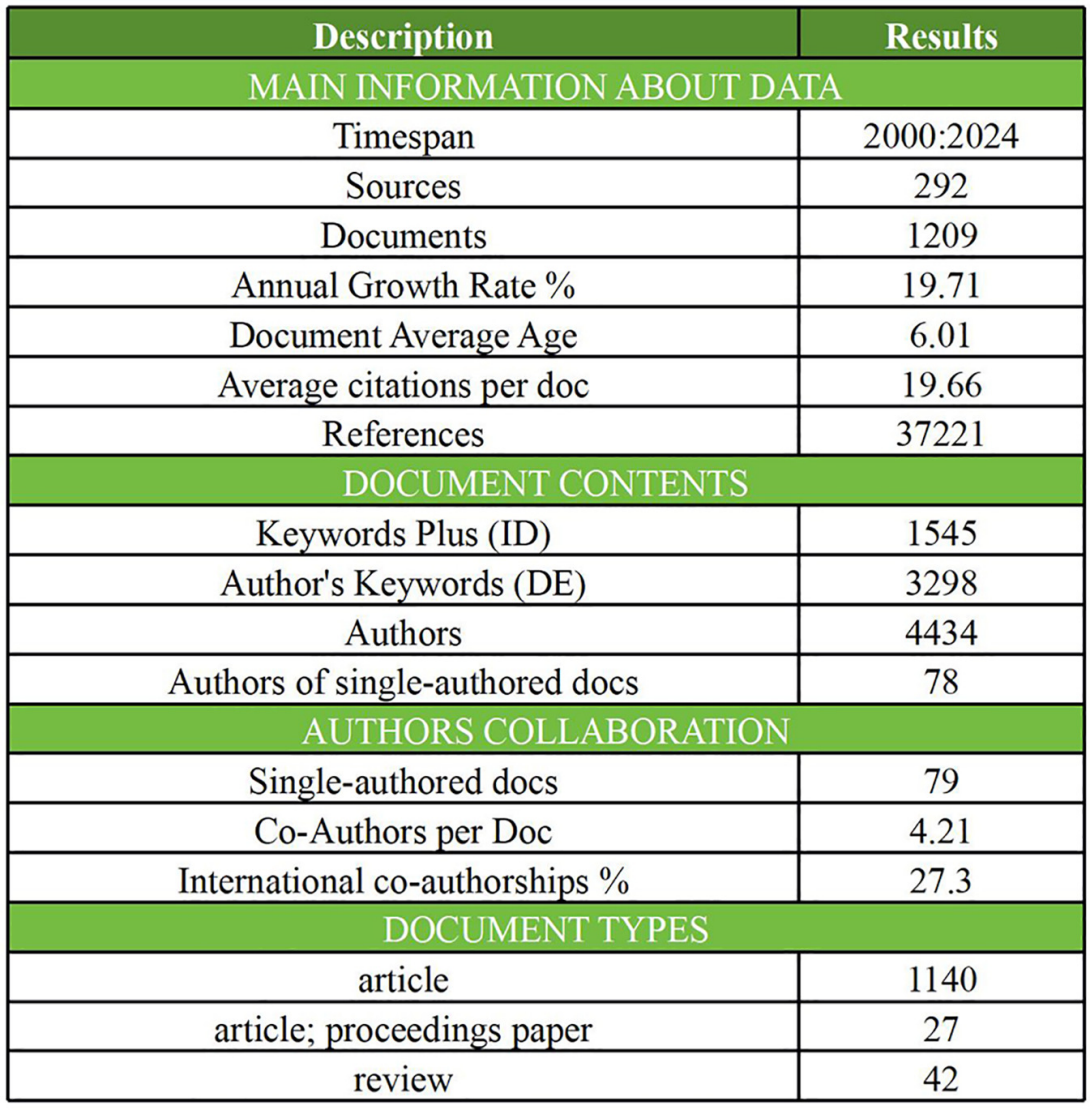
Overview of sports computer vision research.

Figure 2 presents an overview of the literature data, which includes documents authored by 4,434 researchers from 70 countries, published across 292 sources, and collectively cited 37,211 times. Among these, 78 documents are single-authored. On average, the documents are 6.01 years old, with an annual growth rate of 19.71%. The average number of co-authors per document is 4.21, and international co-authorships account for 27.3% of the total. The subsequent sections will provide a detailed analysis of the dataset, focusing on temporal trends, knowledge structures, geographical distributions, journal contributions, and collaboration patterns.

### 3.1 Time series features

**Figure 3:**
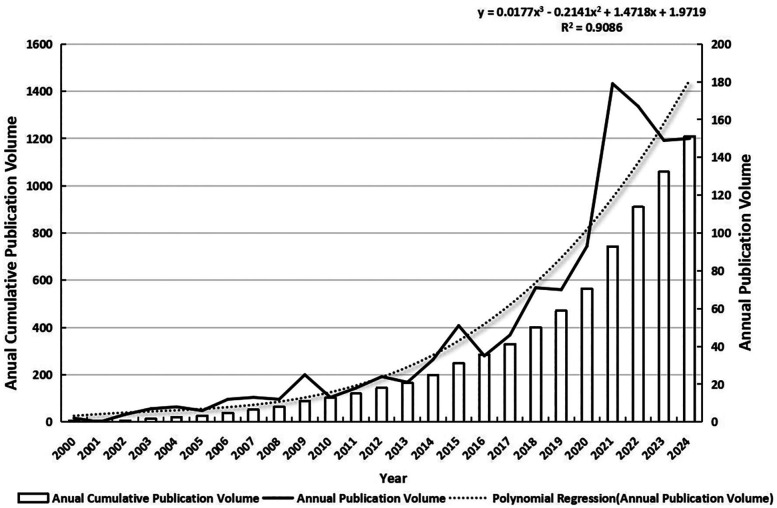
Paper publication trend chart.

Figure 3 presents the trends in annual and cumulative publications, along with exponential fitted curves for annual publications in SCV since the 21st century. The field has seen a sharp increase in publications since 2015, with 1,011 articles published by the end of 2024, representing 83.62% of the total output (2000–2024). This highlights that the majority of the field's development has occurred within a concentrated period of approximately 10 years.

Will the field remain hot, and will the publications grow further in the coming years? Based on the results of the polynomial regression model, the forecasts of the number of publications for the next three years are as follows: approximately 206 predicted publications in 2025, 233 in 2026, and 263 in 2027. This result is relatively reliable (R^2^ = 0.9086), indicating that the model can explain approximately 90.86% of the data variability, successfully capturing most of the trends in the data. Therefore, research in this area will likely continue to increase in the next three years, as it has in the past, and young scholars may consider this area as a promising research direction. However, it should be noted that polynomial regression, while fitting the current data well, may be prone to overfitting and may not fully capture potential fluctuations or external factors that could influence future publication trends. Additionally, the model's uncertainty was not quantified through confidence intervals or prediction intervals, which limits the robustness of the forecasts. Future studies could benefit from incorporating methods such as bootstrapping or time series analysis to better assess prediction uncertainty.

In addition, the field of SCV is not a “flash in the pan” and has a deep knowledge base in the literature. According to annual cumulative publication data, the literature in this field is increasing in thickness and has a deep academic foundation. This finding also proves that it is feasible for young scholars to choose this field of study as their long-term research direction.

### 3.2 Characteristics of discipline structure

**Figure 4:**
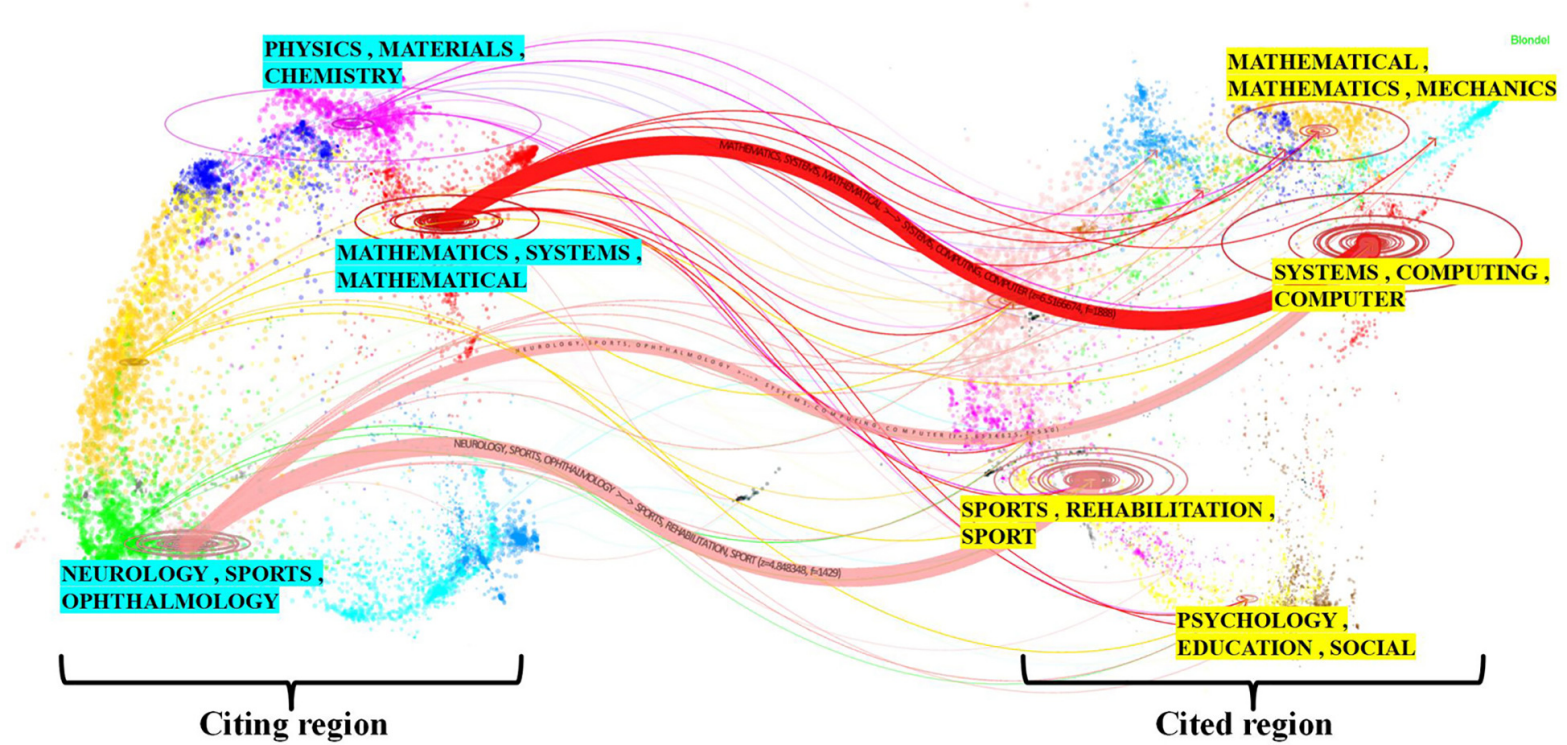
Discipline structure.

Figure 4 represents the disciplinary categories of SCV and the relationships between them. In this figure, the left side depicts the journal group containing the citing literature, representing the primary subject of SCV; the right side corresponds to the journal group of the cited literature, which represents the knowledge domains predominantly cited in SCV.

In addition, the former can be seen as the frontier of research or the area of application, which is mainly concentrated in journal clusters of three types of disciplines:

1. PHYSICS, MATERIALS, CHEMISTRY.
2. MATHEMATICS, SYSTEMS, MATHEMATICAL.
3. NEUROLOGY, SPORTS, OPHTHALMOLOGY.

The latter can be seen as the disciplinary knowledge base of research and is concentrated in four types of disciplinary journal clusters:

1. MATHEMATICAL, MATHEMATICS, and MECHANICS.
2. SYSTEMS, COMPUTING, COMPUTER.
3. SPORTS. REHABILITATION, SPORT.
4. PSYCHOLOGY, EDUCATION, SOCIAL.

Citation trajectories are distinguished by the color of the citing region, and the thickness of these trajectories is proportional to the frequency of citations scaled by the Z-score. Based on the citation trajectories, we can obtain a precise picture of how knowledge flows between the various clusters and Table 2 summarizes these paths with the names of the citing region and the cited region.

**Table 2:** Citation trends at the domain level.

Citing region	Cited region	Z-score
Mathematics, Systems, Mathematical	Systems, Computing, Computer	6.517
Neurology, Sports, Ophthalmology	Sports, Rehabilitation, Sport	4.848

Table 2 sorted by the Z-score in descending order (31), we find that there are two outward citation paths in the left citation domain for the journal groups NEUROLOGY, SPORTS, and OPHTHALMOLOGY and that this group is therefore the most dominant citation group. OPHTHALMOLOGY, there are two outward citation paths in this group of journals, so this group is the most dominant group of citing journals. Moreover, when SYSTEMS, COMPUTING, and COMPUTER are used as the knowledge base, MATHEMATICS, SYSTEMS, and MATHEMATICAL have the highest number of citations, with the highest Z-score of 6.517. Further analysis of the chart revealed that the application of SCV not only incorporates knowledge from mathematics and computer science but also effectively utilizes domain-specific prior knowledge. This research area is influenced by literature from SYSTEMS, COMPUTING, COMPUTER and SPORTS, REHABILITATION, and SPORT, demonstrating the successful integration of interdisciplinary knowledge. Moreover, research in this field draws upon knowledge from PSYCHOLOGY, EDUCATION, and SOCIAL SCIENCE, and extends its applications to PHYSICS, MATERIALS, and CHEMISTRY. It is evident that this field is influenced not only by scientific and engineering disciplines but also by the humanities. Based on these observations, it can be concluded that SCV represents an interdisciplinary research field that integrates multiple disciplines. For instance, researchers have integrated sports psychology with computer vision techniques to classify and identify the movements of boxers, developing models capable of comprehensively understanding the psychological states of athletes. These models provide strong support for both psychological training and movement classification in boxing (39).

### 3.3 Geo-spatial features

**Figure 5:**
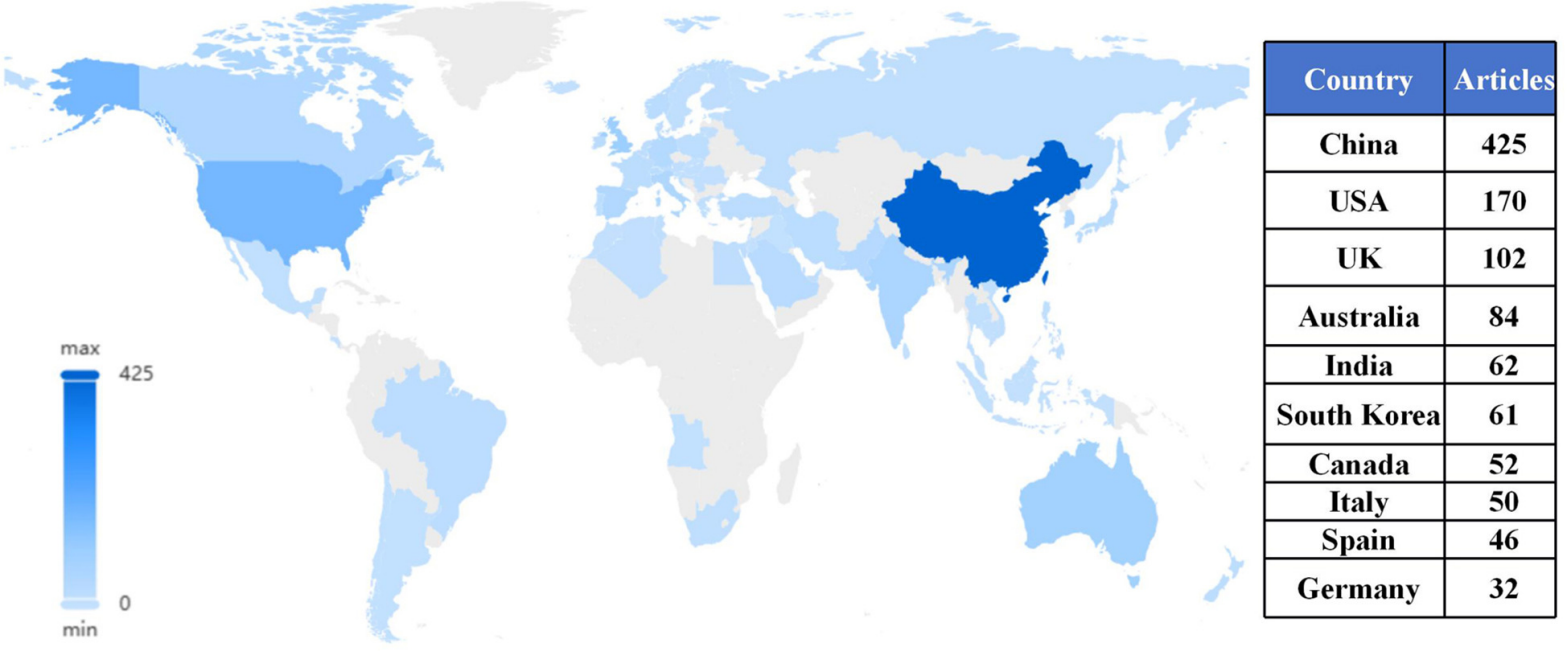
The number of papers published by various countries from 2000–2024.

As illustrated in Figure 5, it presents the geographical distribution characteristics of this field. Research in SCV is most active in China (*n* = 425), the United States (*n* = 170), and the United Kingdom (*n* = 102), with these countries significantly outpacing other regions in terms of research output. This distribution likely reflects strong governmental support for AI research in these leading countries. Policies such as China's “Digital China Development Plan,” the U.S. “American AI Initiative,” and the U.K.'s “National AI Strategy” have played a crucial role in fostering advancements in this field. Additionally, we observe that a considerable number of developing countries, such as Egypt, Saudi Arabia, Turkey, Thailand, the Philippines, and Vietnam, are actively participating in this field. This suggests that the future of SCV research is likely to be a globally collaborative effort, jointly advancing the development of sports technology worldwide.

However, it is regrettable that only a few African countries, such as Angola and South Africa, are currently involved in this research. This limited participation may stem from unequal access to specialized resources, such as high-performance computing infrastructure and private datasets, as well as expertise in data science and machine learning. These constraints could hinder their ability to contribute to global research initiatives. Addressing these disparities will be crucial to fostering a more inclusive and equitable research landscape, enabling all regions to participate in and benefit from advancements in SCV.

### 3.4 Journal distribution features

The distribution of the top 10 journals publishing research in SCV is presented in Table 3 SENSORS leads with 76 published articles, ranking first in terms of output. Furthermore, according to the Journal Citation Reports (JCR) rankings, five of these journals are classified as Q2, four as Q1, and one as Q3, indicating that research in this field is predominantly published in high-impact journals, reflecting the overall high-quality of the work.

**Table 3:** Top journals in sports and computer vision related fields.

Journal	Articles	Quartile	IF (in recent 5 years)
Sensors	76	Q2	3.7
IEEE access	58	Q2	3.7
Multimedia tools and applications	53	Q2	2.9
Journal of strength and conditioning research	27	Q2	3
Computational intelligence and neuroscience	26	Q2	3.877
Neurocomputing	25	Q1	5.5
IEEE transactions on multimedia	23	Q1	8
Journal of science and medicine in sport	23	Q1	3.5
Scientific programming	22	Q3	1.468
Scientific reports	19	Q1	4.3

Notably, among the top 10 journals, only two are specialized in sports science, while the majority are categorized under computer science or multidisciplinary journals. This suggests that the primary platforms for core publications in the field of SCV are rooted in computer science rather than sports science. This trend highlights the interdisciplinary nature of the field, where advancements are driven more by technological innovation than by traditional sports research. Consequently, the integration of computer vision techniques into sports applications appears to be a domain where computer science plays a leading role, with sports science serving as a complementary discipline. This observation underscores the importance of fostering cross-disciplinary collaboration to further advance research and applications in this area.

A key reason why most SCV studies are published in computer science journals rather than sports journals lies in their methodological foundation. These studies typically utilize advanced techniques such as deep learning, object detection, and pose estimation, which align more closely with the evaluation standards and academic readership of computer science journals. By contrast, sports journals tend to prioritize research on training efficacy, theoretical frameworks, and health-related outcomes, and may lack the editorial expertise to assess highly technical AI-based methodologies. Furthermore, interdisciplinary integration between sports science and artificial intelligence is still in its early stages, and dedicated columns or special issues for intelligent sports applications remain scarce in most sports journals.

**Figure 6:**
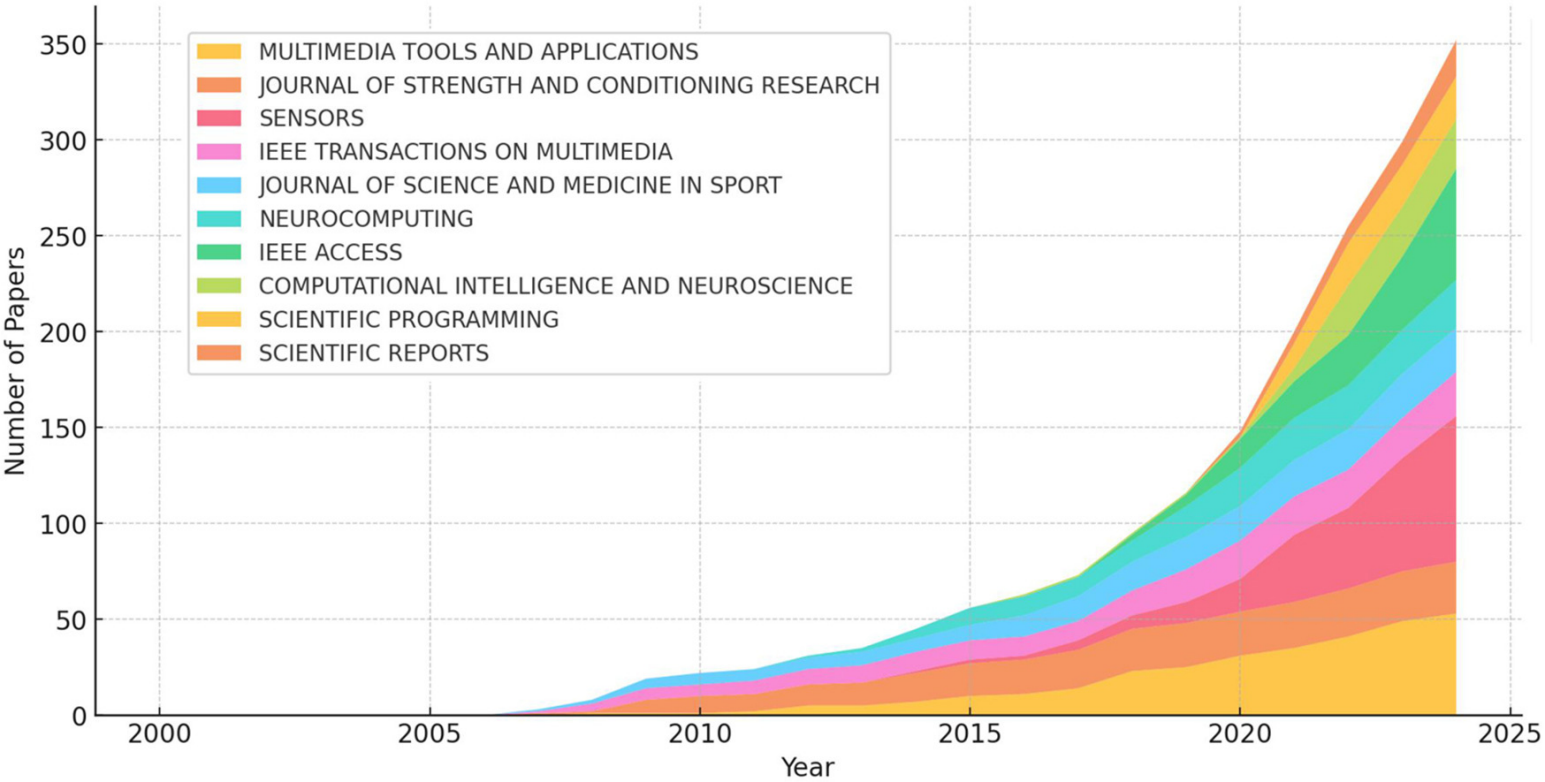
The annual number of journals.

Figure 6 illustrates the temporal trends in the number of publications from the top 10 journals that have published the most research related to SCV. SENSORS experienced significant growth after 2020, with its annual publication volume gradually surpassing other journals to become the leading contributor among the top 10. Similarly, IEEE Access also demonstrated a high publication output, reaching its peak after 2020. As a journal focused on computer and engineering disciplines, the prominence of IEEE Access underscores the application of core computer vision technologies—such as deep learning, object detection, and tracking—in the field of sports science.

Notably, before 2020, SENSORS lagged behind IEEE Access in terms of publication volume. However, after 2020, SENSORS overtook IEEE Access, reflecting a growing emphasis on the application of computer vision in areas such as smart sensors, wearable devices, and motion monitoring systems. This shift highlights the increasing integration of computer vision technologies into sports-related hardware and real-time monitoring solutions, signaling a broader trend toward the convergence of computational methods and sports science. The rise of SENSORS as a leading publication platform further emphasizes the expanding role of sensor-based technologies in advancing SCV research and applications.

### 3.5 Research cooperation features

**Figure 7:**
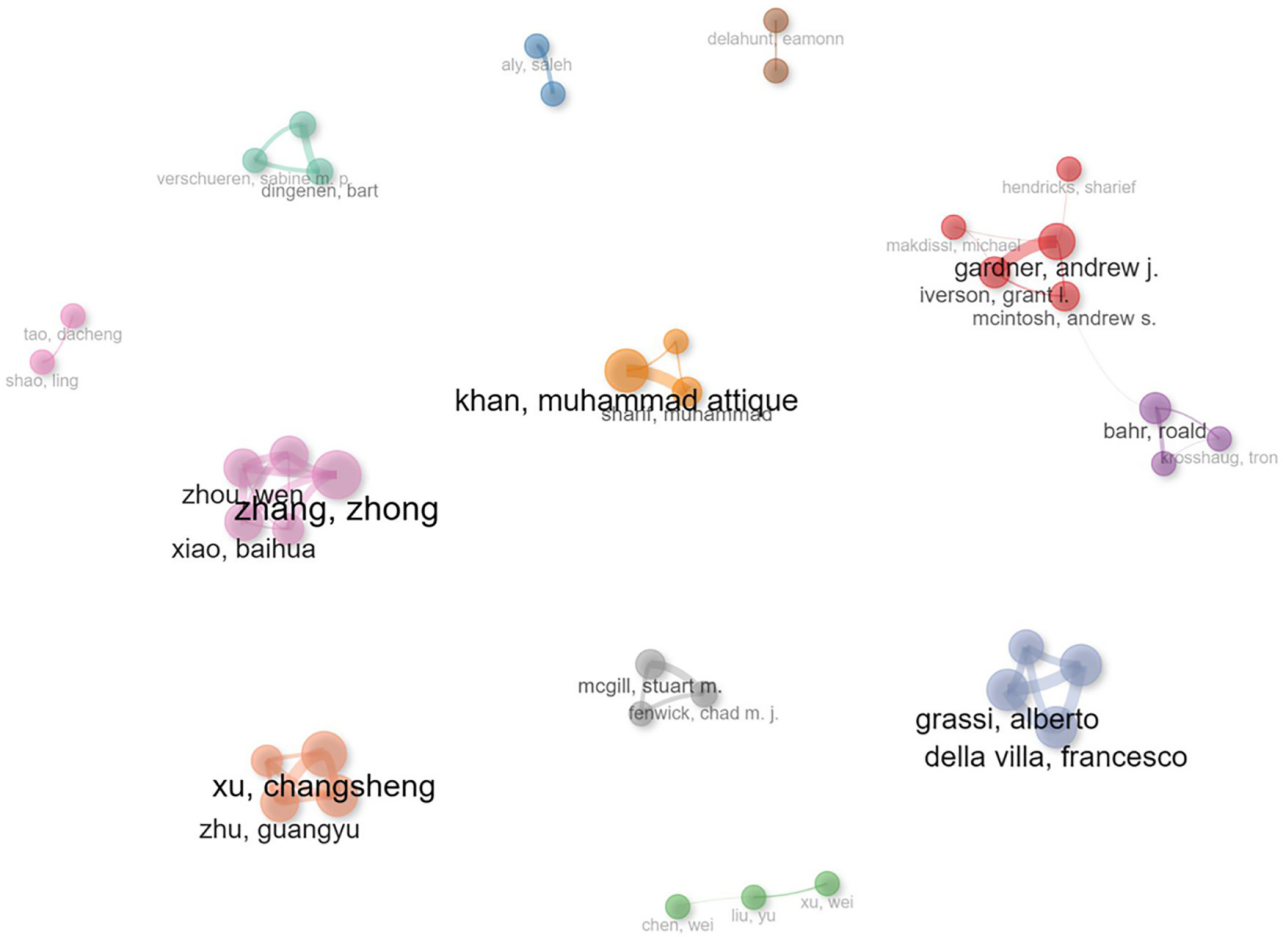
Author collaboration network.

Figure 7 demonstrates the collaborative research characteristics in this field, revealing 12 author clusters with no collaborative links observed among 10 of them, indicating limited research collaboration. Only two clusters, led by Roald and Bahr, and Gardner, and Andrew J, respectively, demonstrate inter-cluster collaboration. This suggests that research teams in the field of SCV are relatively isolated, with collaboration primarily confined to individual teams. Table 4 data further supports this observation: inter-cluster linkages are predominantly mediated by high-betweenness nodes, yet the network as a whole demonstrates marked small-world properties, characterized by strong intra-cluster collaboration and sparse inter-cluster interactions. This “internal cohesion, external isolation” dynamic has yielded a substantial volume of research output. Consequently, this pattern may positively impact researchers' career development and funding opportunities. However, from a sociological perspective, such a “siloed” collaboration model may foster a narrow group identity mentality, potentially leading to exclusivity and a lack of openness. This could pose challenges for scholars seeking to enter the field of SCV research.

**Table 4:** Analysis of node centrality metrics in scientific collaboration network.

Node	Cluster	Betweenness	Closeness	PageRank
Gardner, Andrew J.	1	8	0.083	0.046
Makdissi, Michael	1	0	0.059	0.012
McIntosh, Andrew S.	1	12	0.091	0.025
Hendricks, Sharief	1	0	0.056	0.008
Iverson, Grant L.	1	2	0.077	0.039
Aly, Saleh	2	0	1	0.026
Abdelbaky, Amany	2	0	1	0.026
Liu, Yu	3	1	0.5	0.037
Xu, Wei	3	0	0.333	0.025
Chen, Wei	3	0	0.333	0.014
Bahr, Roald	4	10	0.077	0.033
Krosshaug, Tron	4	0	0.056	0.018
Bere, Tone	4	0	0.056	0.023
Khan, Muhammad Attique	5	0	0.5	0.029
Sharif, Muhammad	5	0	0.5	0.029
Damasevicius, Robertas	5	0	0.5	0.018
Delahunt, Eamonn	6	0	1	0.026
Caulfield, Brian	6	0	1	0.026
Shao, Ling	7	0	1	0.026
Tao, Dacheng	7	0	1	0.026
McGill, Stuart M.	8	0	0.5	0.027
Fenwick, Chad M. J.	8	0	0.5	0.027
Brown, Stephen H. M.	8	0	0.5	0.023
Dingenen, Bart	9	0	0.5	0.027
Staes, Filip F.	9	0	0.5	0.027
Verschueren, Sabine M. P.	9	0	0.5	0.023
Xu, Changsheng	10	0	0.333	0.027
Huang, Qingming	10	0	0.333	0.027
Zhu, Guangyu	10	0	0.333	0.027
Gao, Wen	10	0	0.333	0.023
Della Villa, Francesco	11	0	0.333	0.026
Grassi, Alberto	11	0	0.333	0.026
Zaffagnini, Stefano	11	0	0.333	0.026
Tosarelli, Filippo	11	0	0.333	0.023
Zhang, Zhong	12	0	0.25	0.03
Liu, Shuang	12	0	0.25	0.02
Wang, Chunheng	12	0	0.25	0.026
Xiao, Baihua	12	0	0.25	0.026
Zhou, Wen	12	0	0.25	0.026

Among these clusters, Chinese authors Zhong Zhang and Zhou Wen have collaborated on research topics such as semantic extraction from sports videos and tactical supervision using computer vision techniques (40, 41). Meanwhile, Australian researchers Gardner, Andrew J, and Michael Makdissi have focused on video-based analysis of sports-related concussions and other injuries (42). Similarly, Professor Roald Bahr has made significant contributions to video-based injury analysis in sports such as football and handball, a research area he has pursued since the early 2000s and continues to advance today (43–45). These examples highlight the diverse yet isolated research efforts within the field, underscoring the need for greater interdisciplinary and cross-institutional collaboration to foster innovation and inclusivity in SCV research.

## 4 Discussion

**Figure 8:**
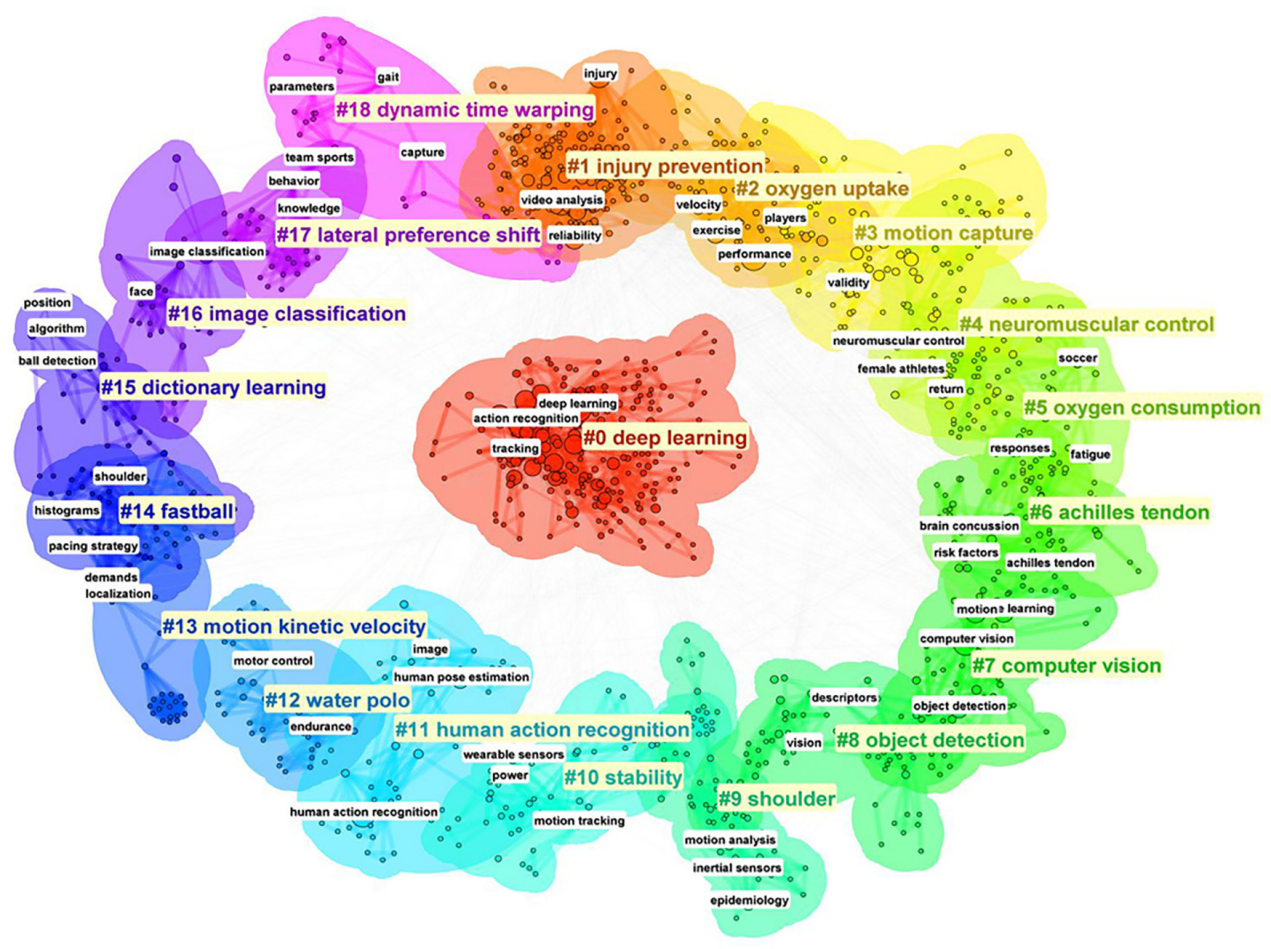
Keywords Clustering map.

Figure 8 presents the keyword clustering results, showing the 18 categories identified in this study. Based on the keyword co-occurrence and clustering analysis of the literature data, this paper summarizes the SCV since the 21st century. These include:

• Sports Skill Enhancement and Technique Optimization (Clusters 0#, 3#, 7#, 11#, 13#, 17#).
• Health Maintenance and Injury Prevention (Clusters 1#, 4#, 5#, 6#, 9#, 10#).
• Physical Fitness Monitoring and Performance Evaluation (Clusters 2#, 5#, 12#, 14#).
• Tactical Analysis and Assisted Refereeing (Clusters 7#, 8#, 15#, 16#, 18#).
• Immersive Event Experience (Clusters 0#, 3#, 7#, 11#).

### 4.1 Sports skill enhancement and technique optimization

This research direction primarily focuses on leveraging SCV technology to analyze athletes' movements and techniques, thereby enhancing the accuracy and efficiency of sports performance and skill development (46). In response to this practical demand, scholars have integrated technologies such as deep learning, motion capture, and motion recognition to analyze athletes' movements and competition videos. This approach ensures scientific precision and refinement in performance analysis, facilitating technical optimization and skill enhancement (47). For instance, motion capture technology has been utilized to measure swimmers' speed, angular velocity, and other kinematic parameters, combined with analyses of swimming posture and fluid resistance effects, to help athletes refine their techniques (48). Additionally, some researchers have explored the application of visual analysis to the nervous system and muscle control, providing interdisciplinary insights to further support movement optimization (49). The impact of SCV technology goes beyond training analysis alone. By integrating multimodal data, intelligent coaching systems have been developed. For instance, an SCV-based AI coaching system can monitor a basketball player's shooting motion in real-time, analyzing factors such as force sequence and shot angle. If deviations from optimal performance are detected, the system can offer immediate feedback through auditory or vibrational cues (50). Another notable example is the China Diving Team's “3D + AI” diving training system, which captures body posture data within 1–2 s for quantitative assessment. These advancements highlight how SCV-based technologies have become a focal point for breakthrough research and practical applications in athlete skill enhancement and technical optimization.

Nevertheless, its application is still mainly concentrated in a few sports and has not yet fully covered the wider sports landscape. Future research should focus on expanding SCV technology to additional sports, particularly martial arts, fencing, surfing, and other specialized disciplines, which deserve more attention, as well as the development of compatible devices and algorithms. In addition, many current motion optimization systems provide real-time feedback on athlete performance through machine learning and image recognition techniques, but the training data of these systems may be biased, especially in terms of gender, race, or body type. This may result in the performance of certain groups not being fairly evaluated. Future research should explore ways to reduce algorithmic bias and ensure that systems can be applied fairly to all types of athletes, especially in cross-cultural and cross-gender sports environments.

### 4.2 Health maintenance and injury prevention

This research direction focuses on utilizing SCV-based technologies for posture analysis, motion capture, and biomechanical assessment to enable injury risk prediction, prevention, and optimized rehabilitation (51). In this field, motion analysis systems must achieve high precision in detecting subtle movement changes, such as gait patterns, swing dynamics, step frequency, step length, and 3D joint angles. Traditional marker-based motion analysis systems, however, require additional markers for data collection, which limits their practicality in routine rehabilitation settings. In contrast, markerless SCV-based techniques enable precise posture monitoring and risk assessment, identifying high-risk movements (e.g., knee inversion, over-rotation) (52), and facilitating the development of rehabilitation protocols through joint position and movement trajectory analysis (53). For instance, multi-angle video analysis can assess the risk of anterior cruciate ligament (ACL) injuries (54). These methods are not limited to competitive sports but also extend to school sports. By capturing students' movement postures and comparing them with incorrect postures in a predefined “video set,” real-time alerts can be issued, and intervention strategies can be automatically generated to reduce injury risks during physical activities (55). Furthermore, in sports rehabilitation, camera systems can monitor patients' limb movements, evaluate rehabilitation training effectiveness in real-time, and provide quantitative references for adjusting training programs (56).

The collection and analysis of personal health data in athlete health monitoring, especially during injury alert and rehabilitation, involves sensitive biological information that may raise the risk of privacy breaches. Data protection measures need to be strengthened in the future to ensure that athletes' data are not misused during collection, storage and analysis. In addition, how to protect privacy when sharing data across platforms, especially for non-professional athletes, will be a key topic. In detail, future research should focus on the following aspects. First, industry data privacy protection standards should be promoted to ensure the security of personal privacy during data collection, storage, and analysis. Sports management organizations, technology companies, and research institutions must work together to develop a systematic data management framework and introduce technologies such as differential privacy and federated learning to achieve secure data sharing and analysis. Second, cross-platform data sharing and applications should be explored under the premise of privacy protection to address the specificity of sports data. Uniform data security specifications and technical standards can not only improve the applicability of SCV technology but also promote international research cooperation and technical exchanges.

### 4.3 Physical fitness monitoring and performance evaluation

This direction is mainly to use SCV to monitor the physical condition of athletes, analyze the functional performance in training, and provide accurate data support for real-time feedback. SCV technology can provide real-time information by combining vision, physiological signals, and biomechanics to systematically detect and evaluate the physical fitness status of athletes (57–59). For example, some researchers have tracked physiological indicators such as the heart rate, respiratory rate, and blood pressure of exercisers in real-time via video analytics for non-contact physiological parameter monitoring techniques (60). Other researchers have analyzed biomechanical metrics such as the joint angle, direction of motion, and speed of movement via multiple Kinect devices and measured their reliability (61). In summary, with the development of physical fitness monitoring technology, athletes' physical fitness status no longer relies solely on a single source of data but rather on the fusion of multiple data to achieve a comprehensive assessment, which helps to reveal the locomotor mechanisms of complex movements.

Currently, data related to physiology and mechanics collected through SCV remains at the stage of manual analysis. In the future, it will be essential to develop an integrated SCV-based system capable of automated detection and multi-modal data analysis, thereby achieving a fully automated and systematic framework for physical training data analysis. More specifically, future research should prioritize the development and refinement of automated methods for analyzing multimodal data. For instance, multi-channel signal processing techniques could be utilized to enable simultaneous analysis of physiological signals and motion imagery, facilitating real-time assessments of sports performance, fatigue monitoring, injury prediction, and training optimization. By minimizing reliance on manual intervention, future systems could significantly enhance the efficiency and timeliness of data processing, ensuring that athletes' movement states are accurately and promptly monitored while reducing the potential for human error. This would ultimately lead to improved health and safety outcomes for athletes.

### 4.4 Tactical analysis and assisted refereeing

This direction focuses mainly on providing automatic detection of key events in the game (62), which can help coaches conduct tactical analysis and assist referees in making accurate penalties. For example, in soccer matches, a multi-view camera system can be utilized to track players' positions and movements accurately and generate top views to simulate tactical maps, thus helping coaches formulate tactical strategies (63). Some researchers have further developed tactical pattern detection algorithms that can identify and classify specific tactical moves in a game, thus helping coaches develop targeted defensive or offensive strategies (64). Some even created a highly automated SCV-assisted volleyball tactical prediction system that learns the historical data and rules of volleyball matches through a combination of CNNs and recurrent neural networks (RNNs), predicts the team's tactical preferences and potential scoring opportunities, and provides real-time tactical suggestions to the coaching team (65). Moreover, the fairness of sports competitions largely depends on accurate judgments, and traditional judgments are prone to errors owing to the limitations of the human eye's field of vision, reaction speed, and occlusion problems (66). The empowerment of SCV technology enables this problem to be solved, such as the development of intelligent referees such as Hawk-Eye, which uses multiple high-speed cameras to capture and process images and relies on a video playback system to place the three-dimensional analog trajectory of the ball on the screen (67, 68). Therefore, the Hawk-Eye system can clearly and accurately determine the landing point of the ball, solve the ambiguity of factual difficulties under the limitations of the naked eye, and better assist the referee in making precise judgments. In addition, VAR in soccer is also a major application that incorporates CV to detect key events (goals, penalties, red and yellow cards, and offsides) in soccer, which has achieved good results, increasing the accuracy rate of key penalties in the English Premier League from 82%–96% (69). Researchers have subsequently attempted to migrate it to the amateur game by constructing the SoccerNet dataset and developing a Video Assistant Referee System (VARS) for automated penalty decisions (70, 71).

In summary, SCV-based video tactical analysis and video assistant referees have become essential components of modern sports competitions. However, both tasks continue to be limited by high computational demands and costly hardware. To overcome these challenges, future research should focus on enhancing algorithmic efficiency and reducing both technical and financial barriers, facilitating the broader adoption of these technologies in small-scale and amateur events. Specifically, lightweight deep learning approaches such as TinyML should be utilized to reduce model complexity, enabling deployment on low-cost hardware. The integration of edge computing can minimize data transmission latency by processing data locally on devices, while adaptive frame rate processing can dynamically allocate resources to critical tactical moments, improving the accuracy of real-time analysis. Moreover, SCV systems based on smartphones or consumer-grade cameras, coupled with open-source analytical platforms, should be developed to improve accessibility and scalability. These systems would provide affordable and effective solutions for tactical analysis and referee assistance, making them accessible to smaller events. Beyond cost and efficiency, expanding the application of SCV to a wider range of sports—such as ice hockey, football, gymnastics, boxing, and esports—can enhance the system's adaptability in complex sporting environments. This expansion would include improving capabilities such as object detection, tactical pattern recognition, and motion trajectory analysis. Finally, ethical concerns related to fairness, bias, and data privacy must be addressed. To mitigate automation bias and ensure system reliability, SCV models should incorporate diverse datasets, employ transparent algorithms, and adhere to stringent data protection protocols. These efforts will help maintain the fairness of competitions while ensuring the responsible application of AI in sports.

### 4.5 Immersive event experience

Research in this area aims to enhance the audience's viewing experience by immersing them more deeply in sporting events and allowing them to better experience the atmosphere of the game scene. Traditionally, sporting events are broadcast through fixed camera angles and manually scheduled screen displays, resulting in a relatively passive and monotonous viewing experience. While this approach provides basic live coverage and replays, it lacks interactivity and data-driven depth. To address these limitations, researchers have explored real-time multi-angle video capture and integration, offering an enriched experience for viewers, event organizers, and performers (72). For instance, one study designed a soccer ball detection system comprising three components (ball detection, single-camera detection, and multi-camera detection). This system employs beam method parity to determine the 3D position of the target, enabling accurate real-time tracking and significantly improving the spectator experience (73). Further advancements have merged CV with Augmented Reality (VR)/Virtual Reality (AR) technologies to reconstruct playing fields and generate 3D perspectives, allowing viewers to observe the game from any angle and enhancing immersion (74). A notable example is Intel's demonstration at the 2024 Paris Olympics, where athlete performances were captured and transformed into 3D models, providing viewers with intuitive competition analysis (75). Additionally, some researchers have integrated game data visualization into this framework. For example, monocular devices have been developed to embed real-time statistical visualizations into the viewing experience. As the game progresses, real-time statistics are displayed on the screen, enhancing viewers' understanding and engagement with the event (76). These innovations demonstrate how SCV technology has revolutionized traditional sports viewing, transforming it into a more interactive, immersive, and data-rich experience.

Currently, most research focuses on enhancing the viewing experience for general audiences, with relatively limited attention given to improving the experience for special populations, such as individuals with disabilities or social anxiety. This represents a promising direction for future academic inquiry. For hearing-impaired individuals, a real-time sign language translation system could be developed. This system would utilize SCV technology to recognize commentators' speech and convert it into visual captions or sign language animations, improving comprehension of event content. An intelligent caption enhancement system could also be introduced to optimize caption layout, color contrast, and readability, ensuring that hearing-impaired audiences can easily access key information.

## 5 Conclusions

This study conducts a bibliometric analysis of the rapidly evolving field of SCV, offering insights that differ from previous understandings.

From a temporal perspective, the rapid growth in this field since the 21st century has predominantly occurred in the last decade (2015–2024), during which 1,011 articles were published, accounting for 83.62% of the total output. This growth trajectory is expected to continue over the next three years, reflecting the increasing interest in integrating SCV to enhance sports applications.

In terms of disciplinary structure, the field is inherently interdisciplinary, encompassing three frontier disciplines and four foundational disciplines. The frontier disciplines leverage cross-disciplinary knowledge from foundational fields such as sports science, computer science, and mathematics to achieve algorithmic breakthroughs. Simultaneously, they integrate wearable devices and biochemical analysis to improve athletic performance and contribute to human health.

Geographically, China (425 articles), the United States (170 articles), and the United Kingdom (102 articles) are leading the development of this field in Asia, the Americas, and Europe, respectively. In contrast, only a few African countries, such as Angola and South Africa, are currently involved in this research. This limited participation may stem from challenges in accessing datasets and acquiring machine learning expertise, which hinders their ability to contribute to global research efforts. Moving forward, international collaboration and support will be essential to overcome these barriers and ensure that the benefits of computer science advancements are accessible worldwide.

From the perspective of journal distribution, high-impact computer science journals serve as the primary platforms for publications in this field, with five journals ranked in Q2 and four in Q1. Among these, SENSORS is the most productive journal, with 76 articles published, consistently ranking first in annual publication volume since 2020. However, only two of the top 10 journals are classified under sports science, indicating a lack of sufficient attention from sports-focused journals to SCV research. This gap suggests a need for future adjustments, such as establishing dedicated special issues to address this emerging interdisciplinary field.

Regarding research collaboration, two significant issues are evident. First, the 12 author clusters within the collaboration network exhibit a pattern of “internal cohesion, external isolation,” which may foster a narrow group identity mentality and create exclusivity. This dynamic could pose challenges for new scholars attempting to enter the field of SCV. Second, the level of international collaboration remains relatively low (27.3%), highlighting the need for stronger academic partnerships both within and across countries. For instance, the collaboration between China's Baidu Paddle team and the National Institute of Sports Science to develop a “3D + AI” diving training system exemplifies the potential of cross-disciplinary and cross-institutional cooperation. To further promote international and interdisciplinary collaboration in this emerging field, we suggest practical measures such as organizing international symposiums, proposing special issues in relevant journals, and encouraging multilateral research projects that bring together experts from sports science, computer vision, and artificial intelligence.

Finally, keyword clustering reveals five major research themes in SCV: Sports Skill Enhancement and Technique Optimization (Clusters 0#, 3#, 7#, 11#, 13#, 17#), Health Maintenance and Injury Prevention (Clusters 1#, 4#, 5#, 6#, 9#, 10#), Physical Fitness Monitoring and Performance Evaluation (Clusters 2#, 5#, 12#, 14#), Tactical Analysis and Assisted Refereeing (Clusters 7#, 8#, 15#, 16#, 18#), Immersive Event Experience (Clusters 0#, 3#, 7#, 11#). However, limitations exist in niche sports applications, privacy protection, automated data analysis, lightweight modeling, and attention to special populations. Looking ahead, future research should expand application scenarios, strengthen data privacy protections, optimize automated data analysis techniques, and promote lightweight modeling for low-resource environments. Additionally, increasing focus on special populations, such as individuals with disabilities, children, and the elderly, will help enhance the fairness and accessibility of these technologies, ensuring their benefits are widely distributed.

However, this study has some limitations. Specifically, the research was restricted to English-language databases, and Chinese databases such as CSSCI and CNKI were not included in the analysis. Given China's significant role in both artificial intelligence and sports, incorporating Chinese databases is essential for a more holistic understanding of the subject. In future studies, we plan to include Chinese databases and compare the results from both Chinese and English sources, offering a more comprehensive perspective. In addition to language and database selection biases, this bibliometric analysis also faces inherent methodological limitations. First, citation lag may lead to an underrepresentation of recently published yet impactful studies, as bibliometric indicators such as citation counts inherently favor older publications. Second, the clustering algorithms employed by tools like CiteSpace and VOSviewer, while powerful, may not always achieve perfect thematic grouping, occasionally misclassifying studies or overlooking nuanced research intersections. These limitations should be considered when interpreting the results.

## Data Availability

The original contributions presented in the study are included in the article/Supplementary Material, further inquiries can be directed to the corresponding author/s.
